# Infrared Target Detection Based on Joint Spatio-Temporal Filtering and L1 Norm Regularization

**DOI:** 10.3390/s22166258

**Published:** 2022-08-20

**Authors:** Enyong Xu, Anqing Wu, Juliu Li, Huajin Chen, Xiangsuo Fan, Qibai Huang

**Affiliations:** 1 School of Mechanical Science and Engineering, Huazhong University of Science and Technology, Wuhan 430074, China; 2 Dongfeng Liuzhou Motor Co., Ltd., Liuzhou 545005, China; 3 School of Automation, Guangxi University of Science and Technology, Liuzhou 545006, China; 4 School of Electronic Engineering, Guangxi University of Science and Technology, Liuzhou 545006, China

**Keywords:** anisotropy, spatio-temporal filtering, robust principal component decomposition model, infrared target, detection

## Abstract

Infrared target detection is often disrupted by a complex background, resulting in a high false alarm and low target recognition. This paper proposes a robust principal component decomposition model with joint spatial and temporal filtering and L1 norm regularization to effectively suppress the complex backgrounds. The model establishes a new anisotropic Gaussian kernel diffusion function, which exploits the difference between the target and the background in the spatial domain to suppress the edge contours. Furthermore, in order to suppress the dynamically changing background, we construct an inversion model that combines temporal domain information and L1 norm regularization to globally constrain the low rank characteristics of the background, and characterize the target sparse component with L1 norm. Finally, the overlapping multiplier method is used for decomposition and reconstruction to complete the target detection.Through relevant experiments, the proposed background modeling method in this paper has a better background suppression effect in different scenes. The average values of the three evaluation indexes, SSIM, BSF and IC, are 0.986, 88.357 and 18.967, respectively. Meanwhile, the proposed detection method obtains a higher detection rate compared with other algorithms under the same false alarm rate.

## 1. Introduction

The infrared detector has the advantages of all-weather, strong anti-interference ability, and high resolution. They are widely used in target monitoring, space debris detection, medical detection instruments, and automobile driving assistance systems. Therefore, scholars have researched infrared target detection algorithms and have achieved corresponding research results.

The literature mainly detects infrared targets through traditional spatio-temporal filtering methods, machine learning, and deep learning methods. The traditional spatio-temporal filtering method models the background or the target based on the target’s local characteristics and the information in the time and space domains to separate the background and target [1,2,3,4,5]. Li et al. [1] proposed a technique that suppresses the background by improving the anisotropic diffusion function. They also established a new adaptive pipe diameter filtering algorithm to extract the target, which achieved good results. Li [2] combined two filtering techniques to extract the bright and dark targets and increase the targets through local comparison. The Laplacian of Gaussian (log) filter and negative log filter can obtain bright and dark targets. Deng [3] describes the local features of an image through multiscale gray difference, which effectively suppresses the background and enhances the target. Xiong [4] proposed a retrieval technique for infrared target images based on the distinct properties of the background, target, and clutter of the infrared gradient vector field. The target of the infrared image was extracted. Then, the clutter is further eliminated, and the target energy is enhanced through the flux of the infrared gradient vector field. Fan [5] enhanced the target energy through high-order accumulation.The traditional spatio-temporal filtering method is based on the local features of the image to separate the background and the target. In the face of complex environments such as lighting changes, dynamic textures, and weak targets without obvious features, traditional spatio-temporal filtering methods often achieve poor detection results.

The literature has employed deep learning to undertake an in-depth exploration of target realization detection in recent years, owing to its great representation capacity, which can more correctly characterize the properties of the target and further increase the target detection rate [6,7,8]. Wang [6] combined RGB camera data with event camera data, employing a cross attention method and a self-attention mechanism to produce good detection results. Gao [7] combined the resolution of the deep and shallow layers of an image. The authors proposed an end-to-end neural network model df-rcnn using deformation convolution and an ROI pool to overcome a non-perfect detection problem of dense vehicles. Hu [8] proposed a convolution neural network algorithm based on background prior by combining a saliency algorithm and a convolution neural network. Their algorithm uses the region of interest as a prior model through a convolution neural network to obtain good detection results. The deep learning detection approach necessitates many training samples for the model to have strong representation capacity, resulting in a time-consuming system. The pre-trained model parameters are difficult to adapt to when the observation scene changes dynamically with time.

The literature has applied machine learning methods to the target detection problem by transforming it into a convex function optimization problem through compressed sensing, matrix reconstruction, and other methods. Then, the convex function is optimized to obtain the target image [9,10,11,12]. Gao [9] proposed an IPI model, which makes full use of the nonlocal autocorrelation of an image. Their proposed approach divides the image into blocks through a sliding window, transforming the target detection problem into low-rank and sparse matrix restoration to achieve good detection results. Wang [10] constructed a target detection model, focusing on the impact of noise and clutter on the model in an actual scene, which achieved a better detection performance and an optimal solution faster than the original model [9]. Wang [11] established a total variation (TV) regularization term based on the IPI model to constrain the low-rank background for a better detection effect despite a complex edge contour background. Wu [12] proposed a gradient difference regularization factor to further suppress the edge contour in the background, obtaining a better detection effect. Zhou [13] proposed a detection method combining spatial feature map regularization and $l_{1,2}$ norm based on the IPI model; the advantage of this method is to fuse data and features manifold with the help of the graph Laplacian form, deeply explore the geometric information of data and feature space, which is used to achieve further constraint on sparse components, and obtain a better target extraction effect. However, the above method only constrains the background by a simple nuclear norm, which leads to the inability to suppress the strong edge contours when facing a complex background containing many strong edge contours, resulting in a high false alarm rate in the detection results. In order to effectively restore the background to extract the target, Sur Singh Rawar [14] proposed the Total Variation Partial Sum Minimization of Singular Values (TV-PSMSV) model based on infrared patch image nuclear norm minimization to detect the target. This model integrates the total variation model into the background modeling model, achieving the purpose of background suppression and energy enhancement. However, the calculation of total variation will make the restored image excessive reduction and the loss of detailed information of the image, that is not conducive to target detection in complex scenes. In the spatial-temporal features measure model (STFM) proposed by Mu [15], firstly, local grayscale difference model is proposed based on the local information of target imaging to analyze the local information, and then short-term energy aggregation model is proposed based on the local information to enhance the energy of dim and small targets in the local region of interest. Finally, combined with energy enhancement and local information analysis, the ong-term trajectory continuity detection model is proposed to obtain the difference image, and perfect results are achieved. It shows that it is important to detect dim and small targets based on the use of spatio-temporal domain information. Dai [16] proposed an infrared patch tensor (IPT) model, extending the dim and small target detection model from a two-dimensional matrix to a three-dimensional tensor field. Zhang [17] combined the weighted kernel norm and $l_{1}$ norm based on the IPT model to constrain the background. Guan [18] improved [17] by combining the tensor kernel norm with the Laplace function, better approximating the non-convex $l_{1}$ norm. Zhang [19] proposed a non-convex optimization detection method based on $l_{p}$ norm constraint(nolc). Their proposed nolc method strengthens the $l_{p}$ norm sparse item constraint and achieves good detection results. Target detection algorithms based on machine learning do not fully utilize local feature information.To further explore the inter-frame information of sequence images, Sun [20] extends the traditional spatial block tensor model to the spatio-temporal block tensor model, obtains the inter-frame information of images with the help of spatial-temporal TV regularization, and combines the weighted tensor kernel norm to suppress the strong edge contours of the background, and obtains a good background forecasting effect. On the basis of the IPI model, Fang [21] suppresses clutter and edge noise by TV regularization and constrains the non-target using a weighted $l_{1}$ norm, and finally obtains a better target detection effect. However, the total variance regularization factor established by the two methods in papers [20,21] only focuses on the difference between a pixel and its neighboring individual pixels, which only simply achieves the effect of smoothing the background, and this regularization factor is not sufficient to completely describe the complex background information in the face of complex scenes. A target with strong edge contour and noise in the detection process breaks the low-rank background characteristics, resulting in detection results that cannot eliminate the impact of contour and noise.

In summary, the above algorithm target detection effect is not satisfactory when facing the complex background. To overcome the shortcomings of the existing detection methods, we propose a detection method based on joint spatio-temporal filtering and L1 norm regularization. The main contributions of this paper are as follows:

1. A new anisotropic Gaussian kernel diffusion function, which makes full use of the local spatial feature information of the image, effectively suppresses the edge contour of the image background;
2. By combining the time-domain information and L1 norm regularization, the temporal-domain information of the image is used to globally constrain the low rank characteristics of the background, and the L1 norm is used to characterize the sparse characteristics of the target, which effectively suppresses the dynamic background and achieves good detection results;
3. The overlapping multiplier method is used to solve and reconstruct the image to better separate the background and target components.

## 2. Anisotropic Function Description

In recent years, the anisotropic filter function has achieved an outstanding performance in target detection. The detection model first conducts background modeling on the target image to obtain the difference image containing the target and background image after the background modeling is completed and then conducts the target detection by extracting the relevant features of the difference image. However, in the presence of complicated ground backgrounds, the target’s contour is frequently suppressed as the background in identifying big infrared targets due to the constraint of its nuclear diffusion function as an S-shaped curve, resulting in the scene of target detection failure. Therefore, this paper improves the anisotropic kernel diffusion function by constructing a new monotonically increasing kernel diffusion function based on Gaussian filtering to suppress the background of an image. The Gaussian filtering reduces the noise of the image to reflect the real signal better. The Gaussian filtering is integrated into the anisotropy to construct the Gaussian kernel diffusion monotonically increasing model. It can suppress the background and effectively retain the relevant information of the target, laying the foundation for the subsequent target detection.

### 2.1. Preliminary Work

The target detection based on anisotropy, such as the operating model [22], has achieved good results using the pixel gradient and kernel diffusion functions. The algorithm can suppress the edge contour and noise in the image for a good background modeling effect. Therefore, this study applies the advantage of anisotropic background suppression to infrared target detection for background suppression. The theory of the gradient perception of nuclear diffusion function is reproduced for the methods in [22,23]. The relevant nuclear diffusion function and anisotropic detection models are as follows:

(1) $$\left\{\begin{array}{c} C_{1}=1-\exp^{-{\left(\nabla I/k\right)}^{2}}\\ C_{2}=1-\frac{1}{1+{\left(\nabla I/k\right)}^{2}}\\ C_{3}=\frac{1}{1+\exp[-M(||\nabla I||/k-1)]}, \end{array}\right.$$

where $C_{1}$ and $C_{2}$ are the revised kernel diffusion function [23], $C_{3}$ is the kernel diffusion function [22], ∇I is the gradient value between pixels, *k* is the gradient threshold, $C_{3}$ is the larger parameter introduced by *M*. When ∇I approaches 0, the gradient perception of the diffusion function can be adjusted by adjusting the value *M*, and different pixel gradients can be calculated to achieve the purpose of adaptive background modeling. The background modeling model combined with the anisotropic gradient is as follows:

(2) $$\left\{\begin{array}{c} \Delta f_{U}=f\left(i,j\right)-f\left(i-step,j\right)\\ \Delta f_{D}=f\left(i,j\right)-f\left(i+step,j\right)\\ \Delta f_{L}=f\left(i,j\right)-f\left(i,j-step\right)\\ \Delta f_{R}=f\left(i,j\right)-f\left(i,j+step\right), \end{array}\right.$$

where *f* is the input image, $\Delta f_{U},\Delta f_{D},\Delta f_{L}$, and $\Delta f_{R}$ refer to the gradient difference in the up, down, left and right directions centered on the pixel f(i,j), and step represents the step size between two pixels. The anisotropic filtering function finally defined by combining the gradient in four directions with the kernel diffusion function is as follows:

(3) $$f^{\prime }\left(i,j\right)=\lambda \left[c\left(\Delta f_{U}\right)\times \Delta f_{U}+c\left(\Delta f_{D}\right)\times \Delta f_{D}+c\left(\Delta f_{L}\right)\times \Delta f_{L}+c\left(\Delta f_{R}\right)\times \Delta f_{R}\right],$$

where λ represents constant parameter, generally not more than 0.25, (i,j) represents the coordinate position of pixel point; $f^{\prime }\left(i,j\right)$ is the difference result graph, and c(•) shows the background construction calculation under the corresponding kernel diffusion function combined with gradient.

**Figure 1:**
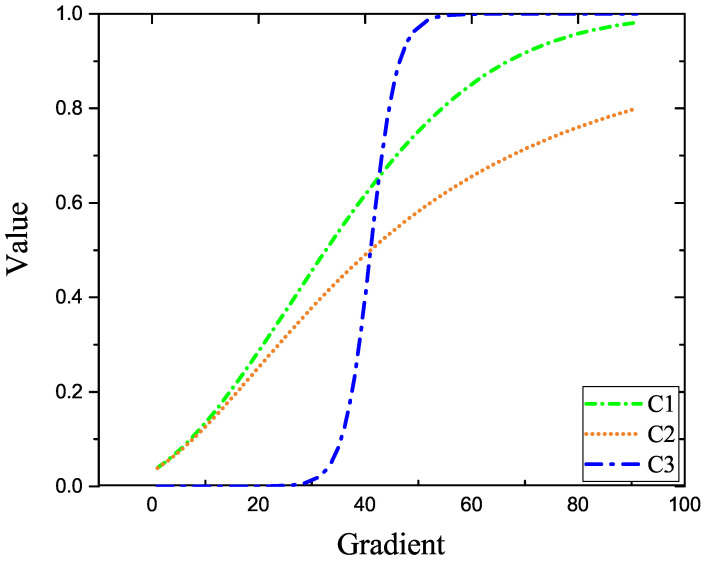
Gradient perception curve of kernel function.

This study simulates the gradient perception of the aforementioned three nuclear diffusion functions to analyze the gradient perception of the aforementioned nuclear diffusion. The corresponding curve is shown in the following Figure 1.

As shown in Figure 1, the gradient perception between pixels of the kernel diffusion function in [22,23] rises gently, indicating that various anisotropic models can retain more edge noise and contour in background modeling the target before detecting the real target. In contrast, the kernel diffusion function [22] has obvious gradient division. The gradient perception is directly separated into fixed gradient values for background modeling by taking advantage of the large gradient difference between the single point target in the image and the neighboring pixels. This method has good applicability to different scenes. It can filter out the targets with the large gradient in the image, contrary to the purpose of large-area infrared target detection. As a result, it is necessary to reconstruct a kernel spread function sensitive to gradient perception. It has a strong inhibition ability to model the image’s background to achieve background suppression while retaining the target’s information to provide conditions for subsequent detection.

### 2.2. New Anisotropic Gaussian Kernel Diffusion Function

Following the analysis of the aforementioned anisotropic kernel function in background modeling, it was discovered that Gaussian filtering achieves the effect of image denoising in target detection, which is consistent with the need to improve the model’s background suppression ability. Therefore, this study considers applying the Gaussian filtering to the anisotropic kernel diffusion function so that the anisotropic model can have a strong gradient perception ability. A strong background suppression ability is utilized to suppress the image’s background, and the target information with a large gradient can be kept to complete the background modeling. The proposed nuclear diffusion model combined with the Gaussian function is as follows:

(4) $$C_{new}\left(\nabla f\right)=\frac{1}{1+M\times e^{-\left(\nabla f/2\right)\times {\left(k\right)}^{2}}}.$$

**Figure 2:**
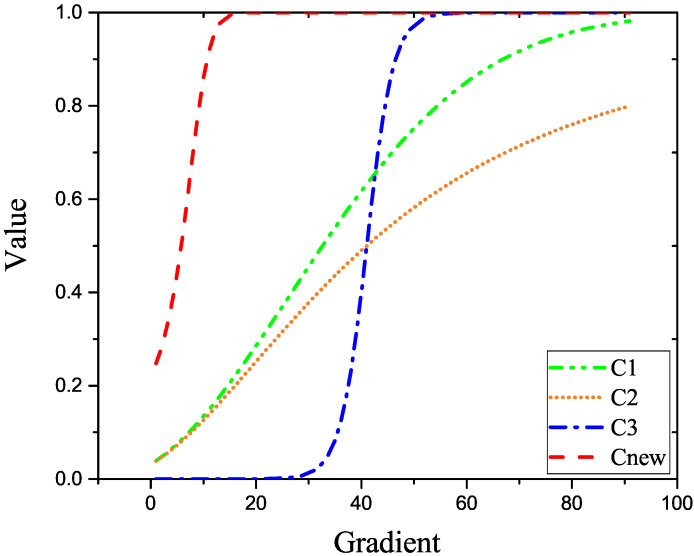
Comparison of gradient perception of kernel diffusion function. All functions were plotted for k=0.1.

In the formula, $C_{new}\left(•\right)$ is introduced into the anisotropic kernel diffusion function, as shown in Equation (4). *M* means that the proposed kernel diffusion function is the introduced constant parameter. Different parameter values can be introduced in different scenes to control the gradient perception to achieve the optimal target detection. ∇f is the gradient value between pixels, and k=0.1 is the gray value threshold. Gradient perception analysis is performed on the created nuclear diffusion function and the aforementioned nuclear diffusion function, and the related gradient perception curve is drawn, as shown in Figure 2.

The constructed nuclear diffusion function is a monotonic increasing function, indicating a strong background suppression ability. The difference between each pixel of the large contour and volume target in the case of infrared target detection is not substantial. The background modeling can be conducted with the same diffusion function value in the target area background modeling to retain that part of the information. Different diffusion function values are used outside the target contour to suppress the background and preserve the infrared target information.

Considering that the target energy diffuses radially to the surrounding during target movement and that the pixel gradients of the target in the up, down, left, and right directions change, each direction has varied background suppression capabilities during background modeling. There is a large difference in the retention of target information. After calculating the pixel gradients in Equation (2), only the average value of the diffusion functions in the four directions in Equation (3) is used as the final filtering result. When the pixel is in the edge contour region, the diffusion function values are large in at least two directions. The diffusion function values of the pixel in the region and the target region will have little difference after simple mean processing. As a result, it is difficult to keep the edge contour region in the background modeling process, resulting in increased edge noise in the distinct images, which is not conducive to extracting the target points. Therefore, this study uses the model result of Equation (5) as final filtering to effectively reduce noise interference on the target signal and achieve the goal of target enhancement. The specific extraction model is as follows

(5) $$\left\{\begin{array}{c} Data=[c\left(\Delta f_{U}\right)\times \Delta f,c\left(\Delta f_{D}\right)\times \Delta f_{D},c\left(\Delta f_{L}\right)\times \Delta f_{L},c\left(\Delta f_{R}\right)\times \Delta f_{R}]\\ B=sort(Data{,}^{\prime }ascend^{\prime })\\ cusm=B(1)+B(2)\\ G=mean(cusm), \end{array}\right.$$

where Data is the set of diffusion function values in each direction, *B* is the set sorted by diffusion coefficients in each direction, cusm is the sum of diffusion functions in the minimum two directions in set *B*, *G* is the mean value of diffusion function values in the minimum two directions, and is the final anisotropic filtering result. The overall process of the model is shown in Algorithm 1.
**Algorithm 1:** Anisotropic Gaussian kernel diffusion function background modeling process.1. Input image;2. Initializing Gaussian anisotropic kernel diffusion function parameters M=20 andk=0.1 in Formula (4) as follow $C_{new}\left(\nabla f\right)=\frac{1}{1+M\times e^{-\left(\nabla f/2\right)\times {\left(k\right)}^{2}}}$.3. Setting anisotropic filtering pixel gradient step in Formula (2) step=4 as follows
$$\left\{\begin{array}{c} \Delta f_{U}=f\left(i,j\right)-f\left(i-step,j\right)\\ \Delta f_{D}=f\left(i,j\right)-f\left(i+step,j\right)\\ \Delta f_{L}=f\left(i,j\right)-f\left(i,j-step\right)\\ \Delta f_{R}=f\left(i,j\right)-f\left(i,j+step\right). \end{array}\right.$$4. Combining Formulas (2) and (4) to calculate the pixel gradient of the pixel in      4 directions, and output the result as $\Delta f_{U},\Delta f_{D},\Delta f_{L},\Delta f_{R}$.5. Using the result in step 4 and the constructed anisotropic filtering model      Formula (5) as follows
$$\left\{\begin{array}{c} \;\mathrm{Data}\;=\left[c\left(\Delta f_{U}\right)\times \Delta f,c\left(\Delta f_{D}\right)\times \Delta f_{D},c\left(\Delta f_{L}\right)\times \Delta f_{L},c\left(\Delta f_{R}\right)\times \Delta f_{R}\right]\\ B=\mathrm{sort}\left(\;\mathrm{Data},\;\;\mathrm{ascend}{\;}^{\prime }\right)\\ \;\mathrm{cusm}\;=B\left(1\right)+B\left(2\right)\\ G=\;\mathrm{mean}\;\left(\;\mathrm{cusm}\;\right). \end{array}\right.$$6. Finish background modeling and output the Difference diagram as *G*.7. end

## 3. The Proposed Detection Model

After relevant scene experiments, the proposed anisotropic Gaussian kernel diffusion filter effectively models most of the background. However, in the process of infrared target detection, it becomes impossible to suppress the dynamic background components by merely employing the spatial domain of the image. To further constrain the low-rank characteristics of the background, an infrared target inversion model combining time domain information and $l_{1}$ norm regularization was proposed. The following focuses on the construction and solution processes of the model. The literature [9] proposes a robust principal component analysis model, which has the following expressions:

(6) $$\begin{array}{c} \min rank\left(B\right)+\lambda {∥T∥}_{0}\\ s.t.B+T=D, \end{array}$$

where *B*, *T* and *D* represent low-rank, sparse, and original matrix, respectively, and λ represents sparse weight. Equation (6) is an NP-hard problem. Therefore, we use the kernel norm to replace the rank of the matrix and the $l_{1}$ norm to approximate the $l_{0}$ norm. The model obtained after the replacement is as follows [10]:

(7) $$\begin{array}{c} \min{∥B∥}_{*}+\lambda {∥T∥}_{1}\\ s.t.B+T=D, \end{array}$$

where ${∥•∥}_{*}$ represents the kernel norm of the matrix, ${∥•∥}_{1}$ represents the $l_{1}$ norm of the matrix, and λ represents the sparse weight. For the infrared target detection model, this study uses the overlapping multiplier method to solve [24]. The augmented Lagrange function of Equation (7) is:

(8) $$L_{A}\left(B,T,Y,\gamma \right)={∥B∥}_{*}+\lambda {∥T∥}_{1}+\langle Y,D-B-T\rangle +\frac{\gamma }{2}{∥D-B-T∥}_{F}^{2},$$

where $\langle •\rangle$ is the inner matrix product, γ is the penalty parameter, and *Y* is the Lagrange operator. The overlapping direction multiplier technique sets one parameter in the model, uses the objective function to minimize the other parameters, and then iterates to find the best solution for the entire model. Update *B* according to Equation (8). In k+1 iterations, *B* can be expressed as:

(9) $$\begin{array}{c} B^{k+1}\leftarrow \arg\;\min L_{A}\left(B^{k},T^{k},Y^{k},D\right)\\ =\arg\;\min{∥B^{k}∥}_{*}+\frac{\gamma }{2}{∥D-B^{k}-T^{k}+\gamma^{-1}Y^{k}∥}_{F}^{2}. \end{array}$$

This problem can be solved using the singular value threshold method [25],

(10) $$B^{k+1}=SVD_{\frac{1}{\gamma }}\left(D-B^{k}-T^{k}+\gamma^{-1}Y^{k}\right),$$

where $SVD_{\tau }\left(•\right)$ is a singular value threshold operator, which is defined as follows

(11) $$\begin{array}{c} SVD_{\tau }\left(Y\right)=Udiag\left[{\left(\sigma -\tau \right)}_{+}\right]V^{T}\\ {\left(\sigma -\tau \right)}_{+}=\left\{\begin{array}{c} \sigma -\tau \sigma >\tau \\ 0\;\;\;otherwise. \end{array}\right. \end{array}$$

Update *T* according to Equation (8). In k+1 iterations, *T* can be expressed as:

(12) $$\begin{array}{c} T^{k+1}\leftarrow \arg\;\min L_{A}\left(B^{k},T^{k},Y^{k},D\right)\\ =\arg\;\min\lambda {∥T^{k}∥}_{1}+\frac{\gamma }{2}{∥D-B^{k}-T^{k}+\gamma^{-1}Y^{k}∥}_{F}^{2}, \end{array}$$

where can be solved using the following operator

(13) $$T^{k+1}=Th_{\frac{\lambda }{\gamma }}\left(D-B^{k}-T^{k}+\gamma^{-1}Y^{k}\right),$$

where $Th_{\varepsilon }\left(•\right)$ is the threshold operator, and the definition is as follows:

(14) $$Th_{\varepsilon }\left(w\right)=\left\{\begin{array}{c} w-\varepsilon w>\varepsilon \\ w+\varepsilon w<\varepsilon \\ 0\;\;\;otherwise. \end{array}\right.$$

Update *Y* according to Equation (8). In k+1 iterations, *Y* can be expressed as:

(15) $$Y^{k+1}=Y^{k}+\gamma \left(D-B^{k}-T^{k}\right).$$

For the γ in Equation (8), the following formula is used to update the iteration process:

(16) $$\gamma^{k+1}=c\gamma^{k},$$

where *c* = 1.5, which is a constant. In the model solution, we define the error tolerance factor, which controls the error between the background, target, and original images. Simultaneously, the number of iterations was limited to prevent overfitting in the model solution. The definition expression of the error tolerance factor is

(17) $$tol=\frac{{∥D-B^{k}-T^{k}∥}_{F}}{{∥D∥}_{F}}.$$

**Figure 3:**
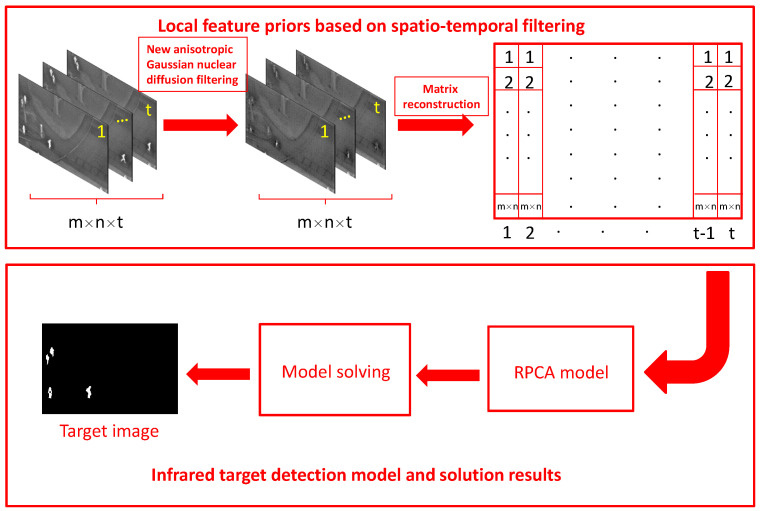
The flow chart of the proposed method.

Thus far, we have proposed a complete model and a solution method. Algorithm 2 shows the algorithm flow chart. The corresponding algorithm flow chart is shown in Figure 3.
**Algorithm 2:** Combined spatio-temporal filtering and L1 norm regularization model.input: image matrix D, parameters λ, c.1. Initialize: $B^{k}=T^{k}=Y^{k}=0$, max_Iter = 500, $tol=5\times 10^{-7}$.while not converged do2. Fixed other parameters and update $B^{k+1}$ by$B^{k+1}=SVD_{\frac{1}{\gamma }}\left(D-B^{k}-T^{k}+\gamma^{-1}Y^{k}\right)$.3. Fixed other parameters and update $T^{k+1}$ by$T^{k+1}=Th_{\frac{\lambda }{\gamma }}\left(D-B^{k}-T^{k}+\gamma^{-1}Y^{k}\right)$.4. Fixed other parameters and update $Y^{k+1}$ by$Y^{k+1}=Y^{k}+\gamma \left(D-B^{k}-T^{k}\right)$.5. Fixed other parameters and update $\gamma^{k+1}$ by$\gamma^{k+1}=c\gamma^{k}$.6. Check the convergence conditions:$\frac{{∥D-B^{k}-T^{k}∥}_{F}}{{∥D∥}_{F}}<tol$ or Iter > max_Iter.7. Update Iter:Iter=Iter+1.end whileOutput: *B*, *T*.

## 4. Results and Analysis

In this section, in order to verify the superiority of this algorithm compared with other algorithms and the robustness of this algorithm in different scenes, the detection effect of this algorithm was compared with seven advanced algorithms in the field of dim and small target detection on eight sequences of representative images, and also draws and analyzes the ROC curves of this algorithm and other algorithms on eight sequences.

### 4.1. Experimental Scenes

Eight scenarios are selected for relevant experiments to effectively reflect the background suppression effect of the Gaussian kernel function constructed in this paper in target detection. The specific datasets are described in the following Table 1.

**Figure 4:**
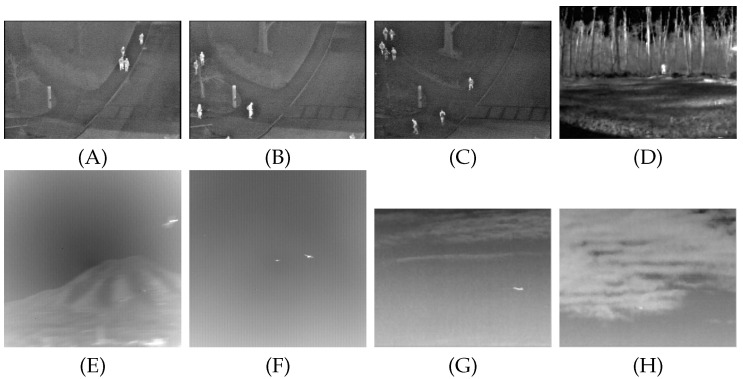
(**A**–**H**) are representative images of eight sequences.

As shown in the table below, to reflect the background modeling effect of the constructed model paper in infrared target detection, eight infrared scenes are selected for the experiment. A representative diagram of the images of the eight sequences is shown in Figure 4.

### 4.2. Background Modeling Results and Analysis

This study employs structural similarity (SSIM), background suppression factor (BSF), and contrast gain (IC) to evaluate and compare the pictures after background modeling to indicate the background modeling effect of the developed nuclear diffusion function in infrared target detection. The specific evaluation indicators are defined as follows [26]

(18) $$\left\{\begin{array}{c} SSIM=\frac{\left(2\mu_{R}\mu_{F}+\varepsilon_{1}\right)\left(2\sigma_{RF}+\varepsilon_{2}\right)}{\left(\mu_{R}^{2}+\mu_{F}^{2}+\varepsilon_{1}\right)\left(\sigma_{R}^{2}+\sigma_{F}^{2}+\varepsilon_{2}\right)}\\ B_{IF}=\sigma_{in}/\sigma_{out}\\ T_{in}=\frac{1}{l\times l}\sum_{x_{g}=-l}^{x_{g}=l}\sum_{y_{g}=-l}^{y_{g}=l}f_{in}\left(i+x_{g},j+y_{g}\right)\\ B_{in}=\frac{1}{l_{1}\times l_{1}}\sum_{x_{g_{1}}=-l_{1}}^{x_{g_{1}}=l_{1}}\sum_{y_{g_{1}}=-l_{1}}^{y_{g_{1}}=l_{1}}f_{in}\left(i+x_{g_{1}},j+y_{g_{1}}\right)\\ T_{out}=\frac{1}{l\times l}\sum_{x_{g}=-l}^{x_{g}=l}\sum_{y_{g}=-l}^{y_{g}=l}f_{out}\left(i+x_{g},j+y_{g}\right)\\ B_{out}=\frac{1}{l_{1}\times l_{1}}\sum_{x_{g_{1}}=-l_{1}}^{x_{g_{1}}=l_{1}}\sum_{y_{g_{1}}=-l_{1}}^{y_{g_{1}}=l_{1}}f_{out}\left(i+x_{g_{1}},j+y_{g_{1}}\right)\\ C_{in}=\frac{|T_{in}-B_{in}|}{|T_{in}+B_{in}|}\\ C_{out}=\frac{|T_{out}-B_{out}|}{|T_{out}+B_{out}|}\\ I=C_{out}/C_{in}, \end{array}\right.$$

where $\mu_{R}$ and $\sigma_{R}$ are the mean and standard deviation of the input image respectively; $\sigma_{RF}$ is the covariance between the input image and the background image; $\varepsilon_{1}$ and $\varepsilon_{2}$ are constants; $\sigma_{in}$ and $\sigma_{out}$ are the mean square deviation of the input image and the difference image, respectively; and $B_{IF}$ is the background inhibitor. $T_{in},B_{in},T_{out},B_{out}$ represent the mean value of different pixel matrices divided by the input image and the output image with the target point as the center, respectively, wherein (i,j) represents the position of the target, $l,l_{1}$ represents different division radii, with values of 1 and 4, respectively; $C_{in}\;and\;C_{out}$ are the contrast of the original image and the difference image, respectively, and *I* is the contrast gain of the input image and the output image.

To reflect the progressiveness of the background modeling model more accurately, seven detection models—Partial Sum of the Tensor Nuclear Norm [17], RPCA [10], Total Variation regulation and Principal Component Pursuit (TV-PCP) [11], Via Nonconvex Tensor Fibered Rank (VNTFR) [27], Asymmetric Spatial-Temporal Total Variation(ASTTV) [28], Self-Regularized Weighted Sparse(SRWS) [29] and anisotropic filtering models [22,23]—are chosen to compare the background modeling. The four detection models selected above and the anisotropic filtering model are used to compare the background modeling. Only the experimental results of background modeling under scene A are shown below, and the rest of the experimental results are detailed in the Appendix A.

**Figure 5:**
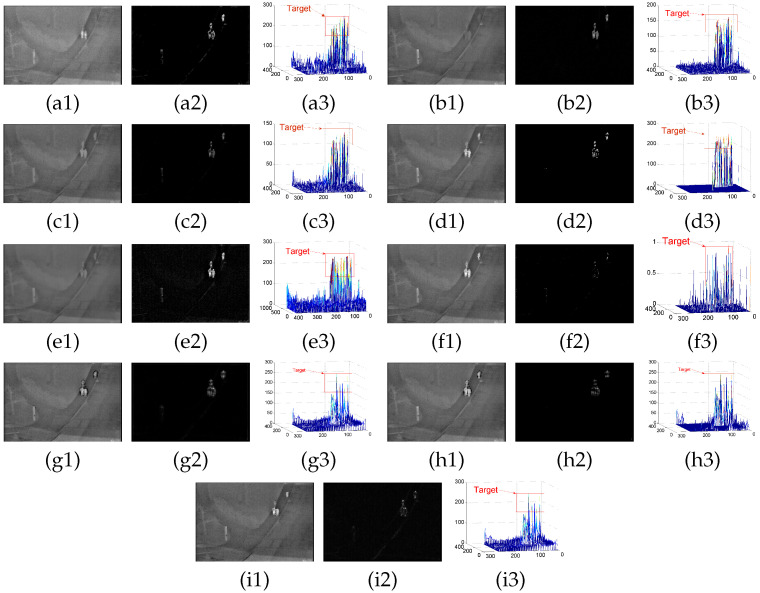
(**a**–**i**) denote the detection results of the PSTNN, RPCA, TV-PCP, VNTFR, ASTTV, SRWS, C2, C3, and the proposed algorithm on sequence A, respectively, where (**a1**–**a3**) denote the background map, the differential map, and the 3D map of the obtained differential map, respectively.

**Figure A1:**
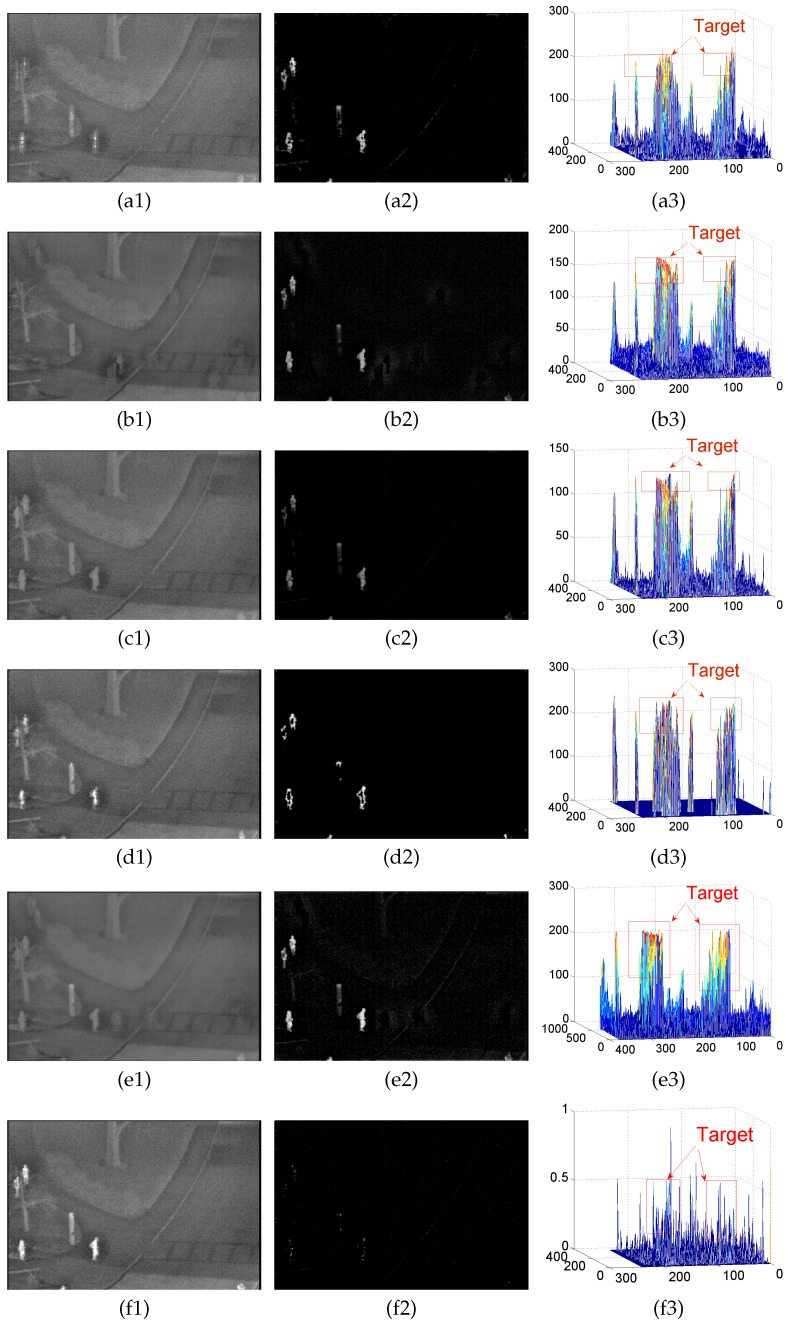

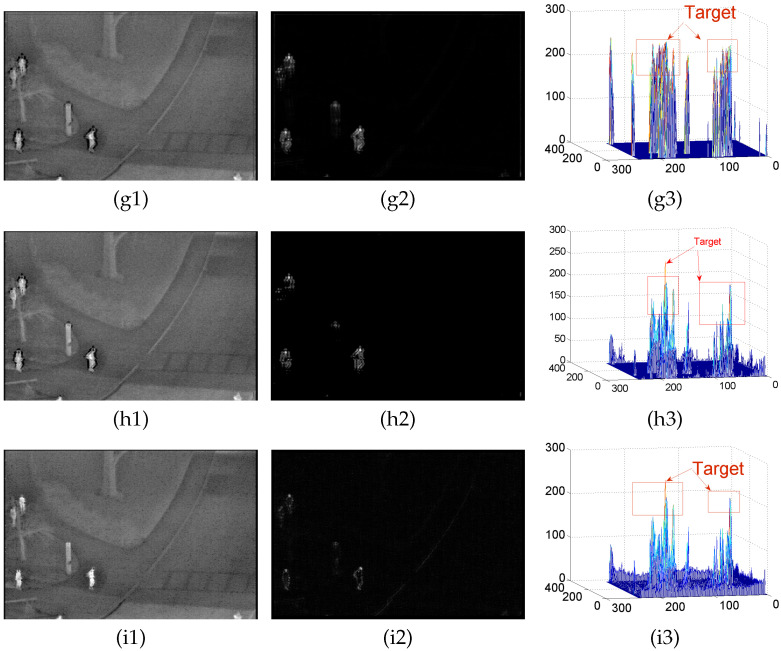
(**a**–**i**) denote the detection results of the PSTNN, RPCA, TV-PCP, VNTFR, ASTTV, SRWS, C2, C3, and proposed algorithm on sequence B, respectively, where (**a1**–**a3**) denote the background map, the differential map, and the 3D map of the obtained differential map, respectively.

**Figure A7:**
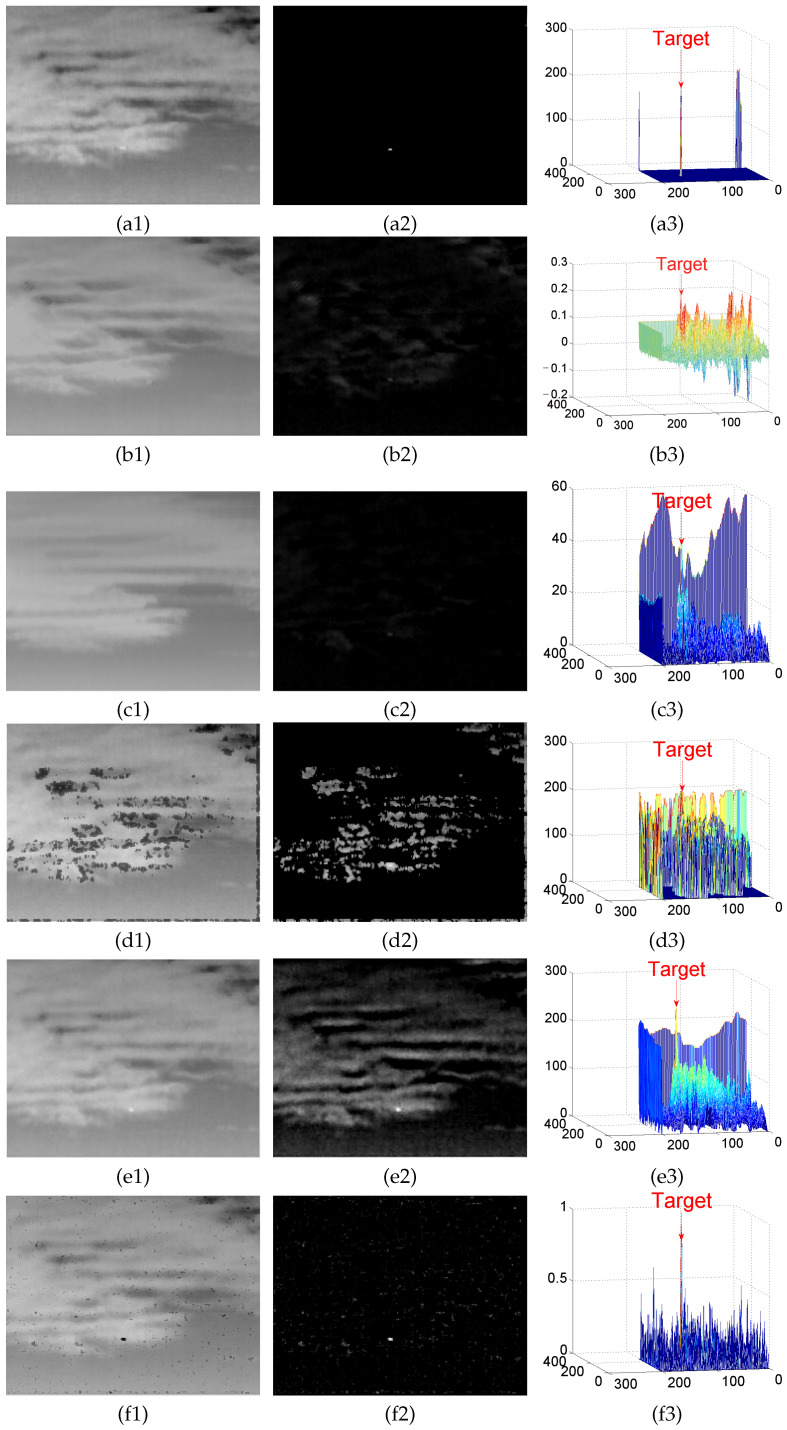

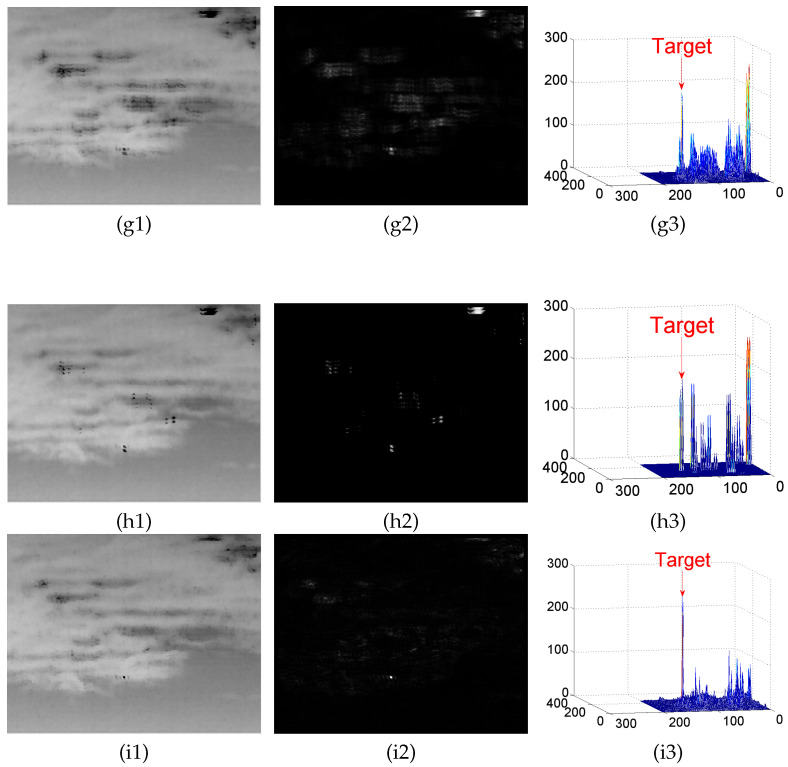
(**a**–**i**) denote the detection results of the PSTNN, RPCA, TV-PCP, VNTFR, ASTTV, SRWS, C2, C3, and proposed algorithm on sequence H, respectively, where (**a1**–**a3**) denote the background map, the differential map, and the 3D map of the obtained differential map, respectively.

The Gaussian kernel diffusion anisotropic filtering model constructed achieves good results in the background modeling of infrared target detection, as shown in the Figure 5 and Figure A1–Figure A7. The proposed model suppresses the background and saves the target signal through the different three-dimensional diagrams, reflecting the algorithm’s feasibility and adaptability. The structural similarity (SSIM), background suppression factor (BSF), and contrast gain (IC) of the aforementioned models after background modeling are evaluated to indicate the originality of the proposed model in the data. The specific experimental data are shown in the Table 2.

The table above shows that the proposed Gaussian kernel anisotropic background modeling model is better than the proposed background modeling model in terms of structural similarity (SSIM), background suppression factor (BSF), and contrast gain (IC). The average structural similarity (SSIM) reached 0.986. The average background suppression factor (BSF) reached 88.357, indicating that the background modeling has a good ability to suppress the background. The average value of the contrast gain IC reached 18.967, which shows that the target information is effectively preserved in the difference map. Simultaneously, it reflects that the Gaussian kernel diffusion anisotropic filter achieved good results in background modeling, satisfying the purpose of background suppression and preserving the target signal. It also shows that the constructed model has good feasibility and scene adaptability and can meet the requirements of infrared target detection.

### 4.3. Detection Results

To verify the effectiveness of the algorithm, this paper lists and compares the proposed algorithm with the PSTNN, TV-PCP, VNTFR, anisotropic algorithm, ASTTV, SRWS, and RPCA algorithms in eight different scenarios. The detection results of the eight algorithms under scenario A are shown below, and the detection results of the remaining scenarios are shown in the Appendix A.

**Figure 6:**
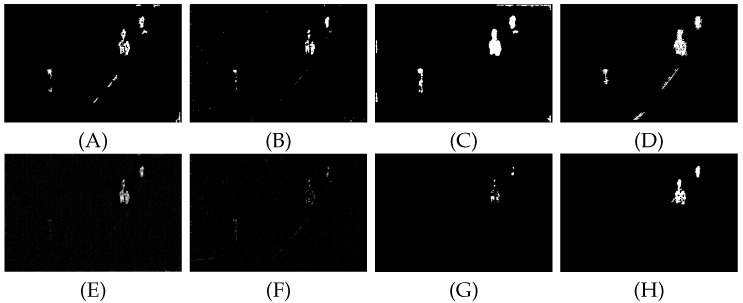
(**A**–**H**) show the detection results of eight algorithms PSTNN, TV-PCP, VNTFR, anisotropy, ASTTV, SRWS, RPCA and propose methods, respectively, under sequence A.

**Figure A8:**
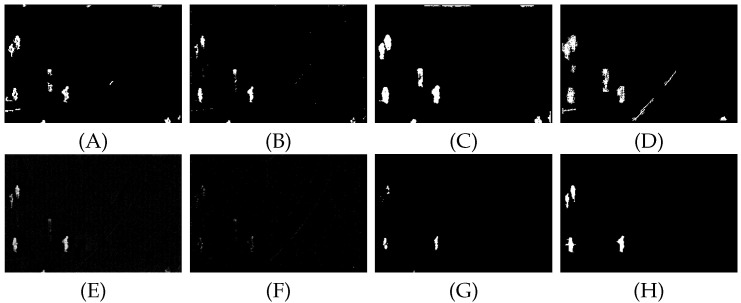
(**A**–**H**) shows the detection results of eight algorithms PSTNN, TV-PCP, VNTFR, anisotropy, ASTTV, SRWS, RPCA and propose method, respectively, under sequence B.

**Figure A10:**
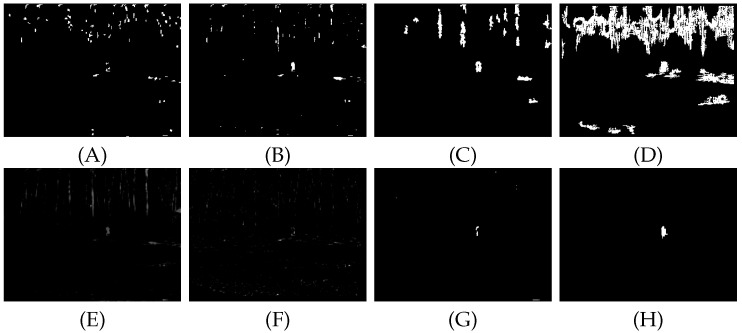
(**A**–**H**) shows the detection results of eight algorithms PSTNN, TV-PCP, VNTFR, anisotropy, ASTTV, SRWS, RPCA and propose method, respectively, under sequence D.

**Figure A12:**
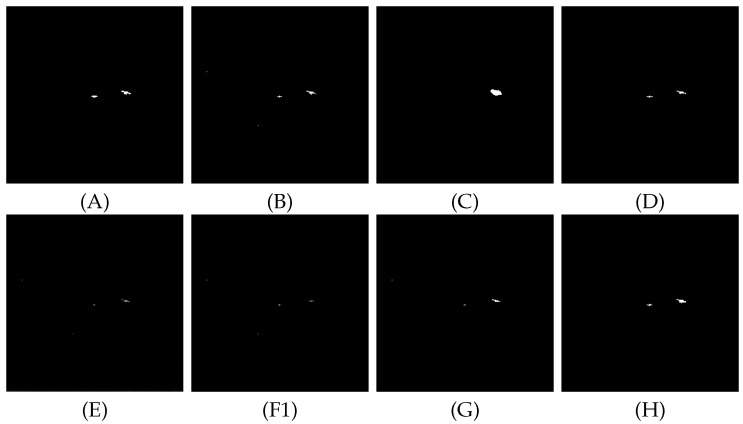
(**A**–**H**) shows the detection results of eight algorithms PSTNN, TV-PCP, VNTFR, anisotropy, ASTTV, SRWS, RPCA and propose method, respectively, under sequence F.

**Figure A14:**
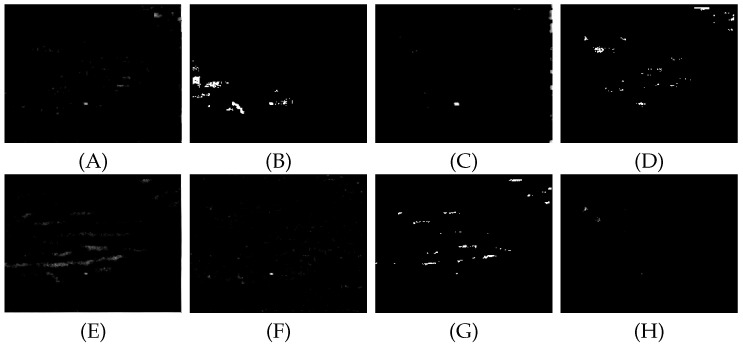
(**A**–**H**) shows the detection results of eight algorithms PSTNN, TV-PCP, VNTFR, anisotropy, ASTTV, SRWS, RPCA and propose method, respectively, under sequence H.

The aforementioned detection results show that the PSTNN algorithm constrains the low-rank components of the background by combining the tensor kernel norm and the weighted L1 norm. The VNTFR algorithm approximates the tensor kernel norm of the logarithmic operator as the tensor fiber rank and then suppresses the noise with the help of the hypertotal variation. However, from Figure 6 and Figure A8, the PSTNN and VNTFR algorithms face the edge contour of a large background with high energy. The background cannot be completely suppressed, resulting in interference signals in the detection results. As shown in Figure A10, when facing the target sunk in the highlighted background, the PSTNN algorithm cannot completely recover the target information, resulting in the loss of target information. The TV-PCP algorithm suppresses the background using a total variation. However, Figure 6 and Figure A8–Figure A14. show that when facing strong noise in the background, the interference of noise cannot be eliminated in the detection results obtained using the algorithm. The anisotropic algorithm describes the background by calculating the difference in each direction between the pixel and the adjacent pixel with the help of the diffusion function. From Figure A10, the anisotropic algorithm cannot suppress the background because the difference between the pixel and each direction is small under a large strong edge contour background. A large amount of background contour noise appears in the final detection result. From Figure 6 and Figure A8–Figure A10, the RPCA algorithm cannot completely recover the sparse components of the model when recovering the target information, resulting in the loss of the target signal. From Figure 6 and Figure A8–Figure A12, it can be found that the SRWS algorithm suppresses the target energy while suppressing the background, resulting in unclear targets in the detection results. Figure 6 and Figure A8–Figure A14. show that the proposed algorithm combines spatio-temporal filtering and the principal component decomposition model. First, the background is suppressed through the improved anisotropic function. Then, the target and background information are further separated through the principal component decomposition model to achieve a good detection effect.

**Figure 7:**
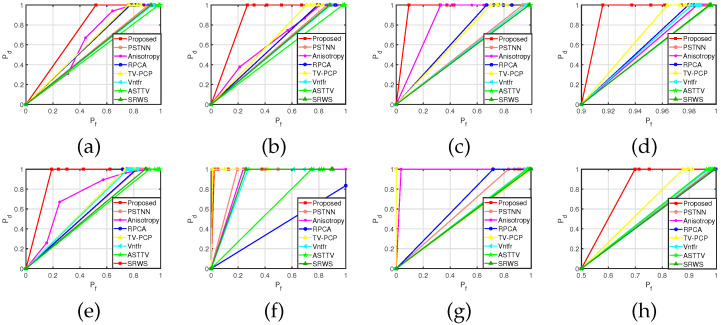
(**a**–**h**) show the ROC curves of eight sequences, respectively.

ROC curves of eight scenarios are drawn to further explore these algorithms’ performances, where the horizontal axis is the detection rate (PD) and the vertical axis is the false alarm rate (PF). According to Formula (19), ntdt is the number of real targets detected and nfdt is the number of false alarm targets detected; NT is the total number of real targets in the image, and NP is the total number of targets detected in the image. The ROC curve is shown in Figure 7.

(19) $$\left\{\begin{array}{c} P_{d}=\frac{NTDT}{NT}\times 100\%\\ P_{f}=\frac{NFDT}{NP}\times 100\% \end{array}\right.$$

As shown in Figure 7a–h, compared with the other seven algorithms, the proposed algorithm achieved a better target detection effect and noise suppression ability on the sequence images based on the robust principal component decomposition model combined with the sequence images of the space–time domain information. As shown in Figure 7a–c, the anisotropic algorithm can effectively separate the background and pedestrians by modeling the background through the description function in the scene with simple background pedestrian detection. The traditional anisotropic description function is not ideal for background modeling when the background is a complex forest, clouds or other scenes with many edge contours, resulting in a high false alarm rate, as shown in Figure 7d,h. Figure 7a–e,h shows that the low-rank characteristics of the background will be destroyed when the background contains more edge contours and strong energy interference noise. The target and background images will be recovered only through the global information of the image. The results of algorithms such as PSTNN, VNTFR, ASTTV, SRWS, TV-PCP, and RPCA are not ideal. Under the same detection rate, the false alarm rate is high. As shown in Figure 7a–h, the proposed principal component decomposition model, which combines the local and global information of the image, shows high robustness in different scenarios.

## 5. Conclusions and Future Direction

### 5.1. Conclusions

In this paper, we propose a target detection method that combines temporal and spatial filtering and the L1 norm to solve the challenge of high false alarm and low target recognition rates in target detection. A new anisotropic Gaussian kernel diffusion function is established to describe the background information. The principal component decomposition model is used to further constrain the low rank features of the background by using the global information of the image. Experimental results show that the proposed method has high SNR, background suppression factor and SNR gain in different environments. The ROC curve shows that the proposed detection algorithm has a higher detection rate and background suppression ability in various sequence scenes.

### 5.2. Future Direction

In the process of model inversion, when facing a scene with more edge contours, only the L1 norm is used to constrain the sparse components of the target. Because the characteristics of the edge contours and the target characteristics are not obvious, the decomposition model contains more false alarm targets. In future work, we can consider building corresponding constraint models to constrain the low rank characteristics of the background and the sparse components of the target at the same time, or combine tensor theory to describe the difference between the target and the edge contour to further highlight the target signal.

## Figures and Tables

**Table 1:** Detailed descriptions of eight real sequences.

Scene	Target Size	Image Size	Number of Scene Frames	Target Motion Description
Scene A	20 × 20	360 × 240	30	Multiple pedestrian movements on campus
Scene B	20 × 20	360 × 240	18	Multiple pedestrian movements on campus
Scene C	20 × 20	360 × 240	18	Multiple pedestrian movements on campus
Scene D	20 × 20	320 × 240	103	Multiple pedestrian movements on campus
Scene E	15 × 15	256 × 256	88	Large aircraft moving in low altitude complex background
Scene F	5 × 5, 3 × 3	256 × 256	599	Large aircraft moving in low altitude complex background
Scene G	7 × 7	256 × 256	30	Large aircraft moving in low altitude complex background
Scene H	5 × 5	256 × 256	28	Large aircraft moving in low altitude complex background

**Table 2:** Background modeling evaluation metrics for different algorithms.

Method	Evaluation Indicators	SeqA	SeqB	SeqC	SeqD	SeqE	SeqF	SeqG	SeqH
PSTNN [17]	SSIM	0.8929	0.9643	0.8007	0.9643	0.9707	0.7127	0.9479	0.9704
	BSF	16.3978	44.5755	14.5276	44.5755	99.984	78.191	81.58	60.2096
	IC	1.7549	4.4669	NaN	4.4669	9.7552	10.77	**46.7006**	**NaN**
RPCA [10]	SSIM	0.8992	0.743	0.8457	0.743	0.9807	0.9814	0.906	0.9035
	BSF	22.7456	17.3042	18.9079	17.3042	51.09	53.682	26.6465	23.1095
	IC	1.7333	1.8644	**52.0959**	1.8644	12.037	7.9779	9.5817	39.1915
TV-PCP [11]	SSIM	0.9418	0.9825	0.9118	**0.9825**	0.998	0.7412	0.9848	0.8935
	BSF	31.1774	55.5603	26.0405	55.5603	157.32	18.67	57.7783	24.4123
	IC	1.2946	2.5605	51.9887	2.5605	**12.429**	3.3153	15.9996	465.7584
VNTFRA [27]	SSIM	0.9733	0.9251	0.9599	0.9251	0.9784	0.8536	0.9281	0.6475
	BSF	19.259	13.9744	17.4709	13.9744	45.428	43.729	19.4073	5.4145
	IC	3.9947	2.7721	NaN	2.7721	20.881	0.9476	8.0377	350.5143
ASTTV [28]	SSIM	0.838	0.8154	0.8533	0.9684	0.928	0.927	0.9386	0.7944
	BSF	12.026	11.953	13.809	22.861	13.07	13.54	11.482	5.8283
	IC	**19.931**	**25.002**	**113.28**	2.3377	**17.1**	11.07	13.4227	3.9597
SRWS [29]	SSIM	0.9707	0.9832	0.9647	0.9965	0.999	0.996	0.991	0.9298
	BSF	40.144	53.383	36.94	118.64	184.2	113	73.5345	25.2542
	IC	3.8509	3.8715	52.096	3.6856	**52.01**	12.9	46.0748	218.8171
C2 [22]	SSIM	0.9096	0.8929	0.8756	0.9594	0.9967	0.9933	0.9922	0.9155
	BSF	22.9123	21.6956	20.2689	33.5829	122.63	86.64	79.1137	19.9491
	IC	1.9262	1.417	16.0295	2.5389	6.501	3.9545	3.7964	81.1303
C3 [23]	SSIM	0.9239	0.9135	0.9339	0.9594	0.9971	0.995	0.995	0.9594
	BSF	24.3321	23.942	26.7567	32.2585	132.31	99.797	98.9412	22.7394
	IC	1.477	1.4636	NaN	4.5367	11.957	7.5737	12.8887	NaN
Proposed	SSIM	**0.9827**	**0.9889**	**0.9685**	0.9819	**0.9989**	**0.9962**	**0.9965**	**0.9774**
	BSF	**53.8529**	**68.4012**	**41.3969**	**57.6583**	**211.49**	**115.01**	**114.9036**	45.1366
	IC	**4.175**	**3.5205**	10.4192	**8.776**	12.375	**12.915**	29.4573	70.1029

**Figure A2:**
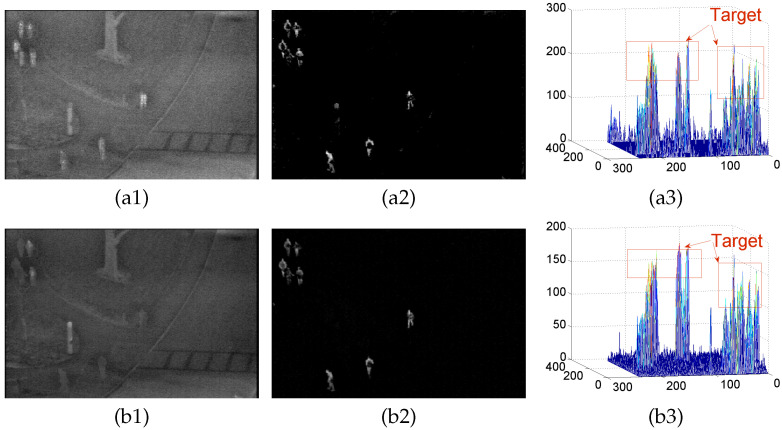

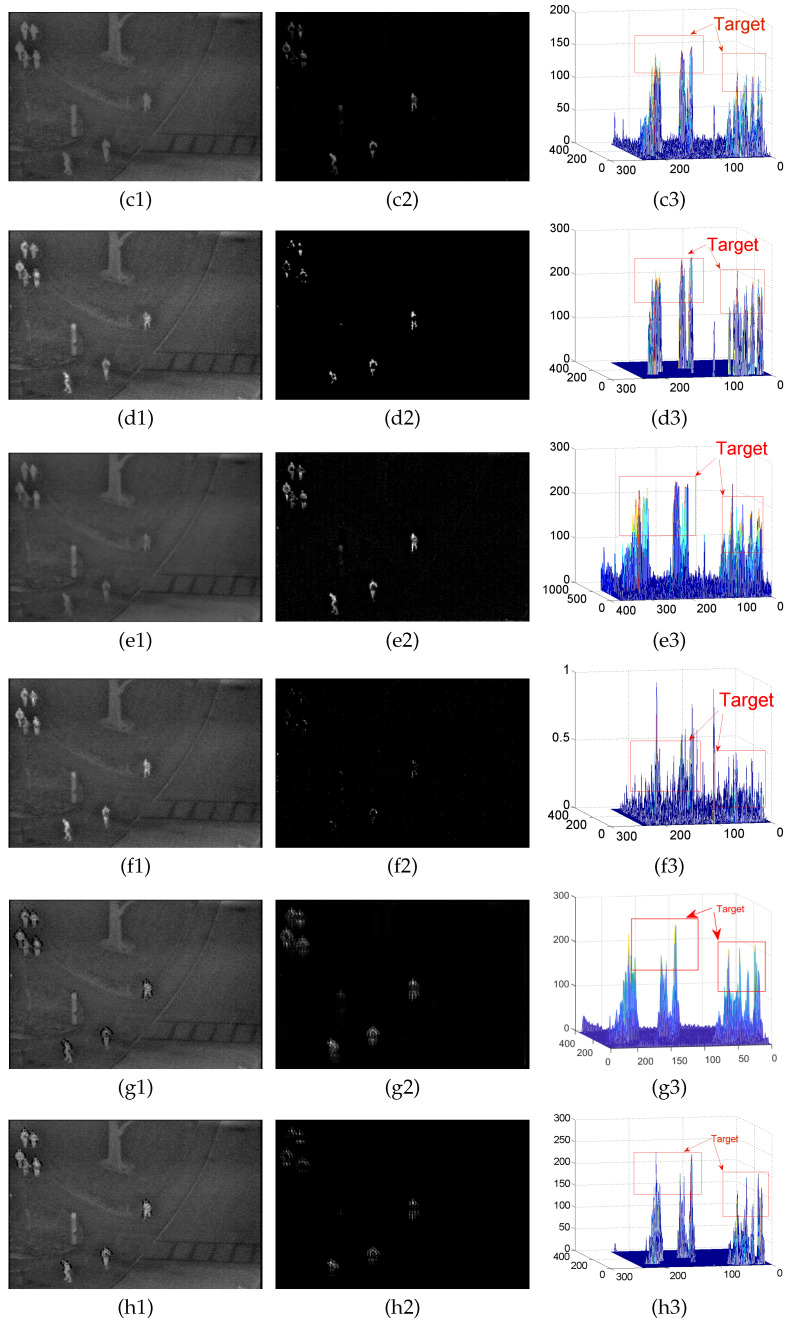

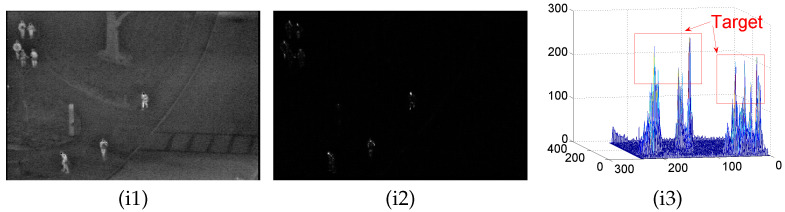
(**a**–**i**) denote the detection results of the PSTNN, RPCA, TV-PCP, VNTFR, ASTTV, SRWS, C2, C3, and proposed algorithm on sequence C, respectively, where (**a1**–**a3**) denote the background map, the differential map, and the 3D map of the obtained differential map, respectively.

**Figure A3:**
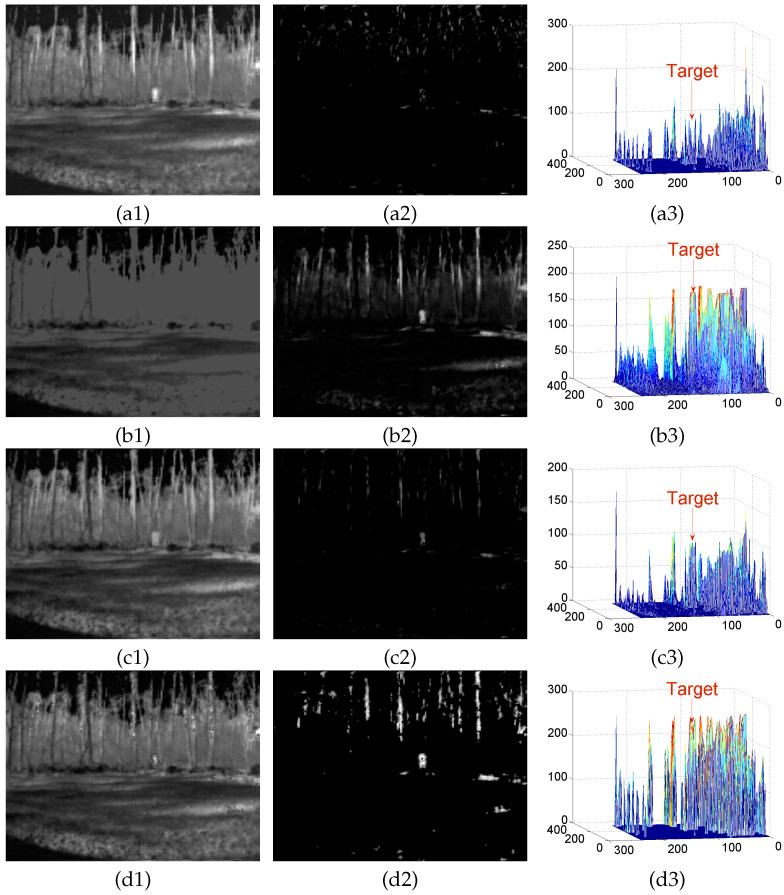

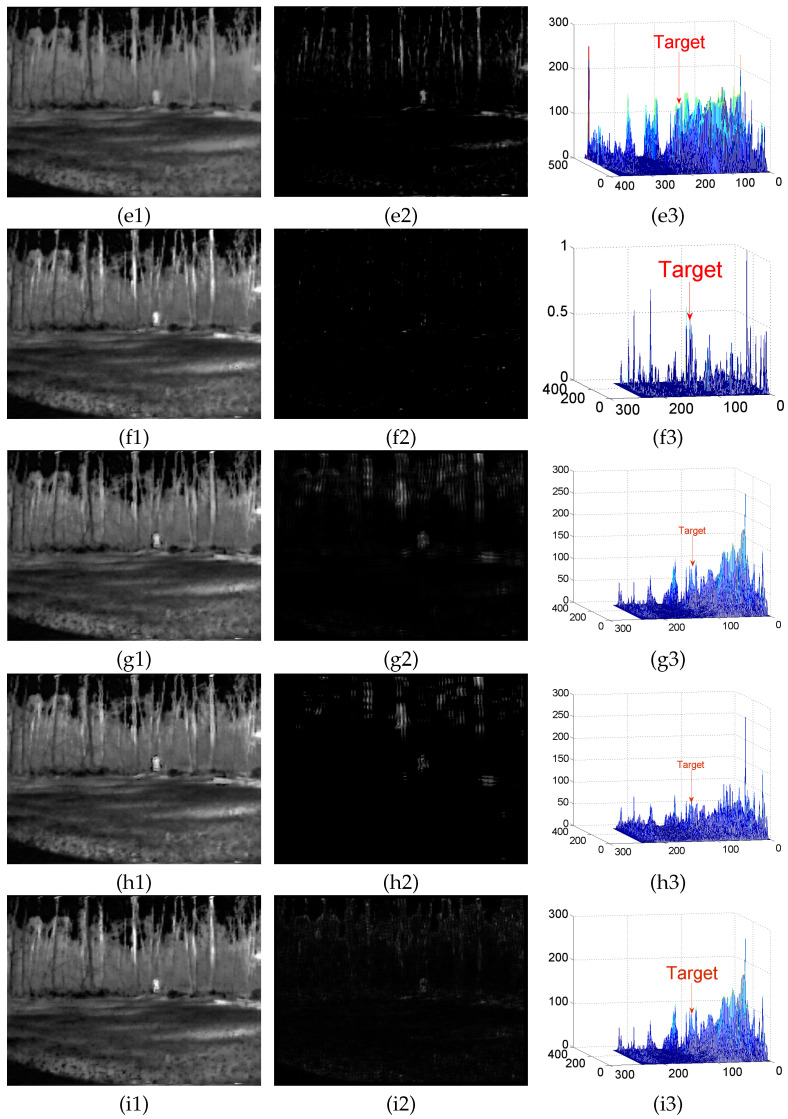
(**a**–**i**) denote the detection results of the PSTNN, RPCA, TV-PCP, VNTFR, ASTTV, SRWS, C2, C3, and proposed algorithm on sequence D, respectively, where (**a1**–**a3**) denote the background map, the differential map, and the 3D map of the obtained differential map, respectively.

**Figure A4:**
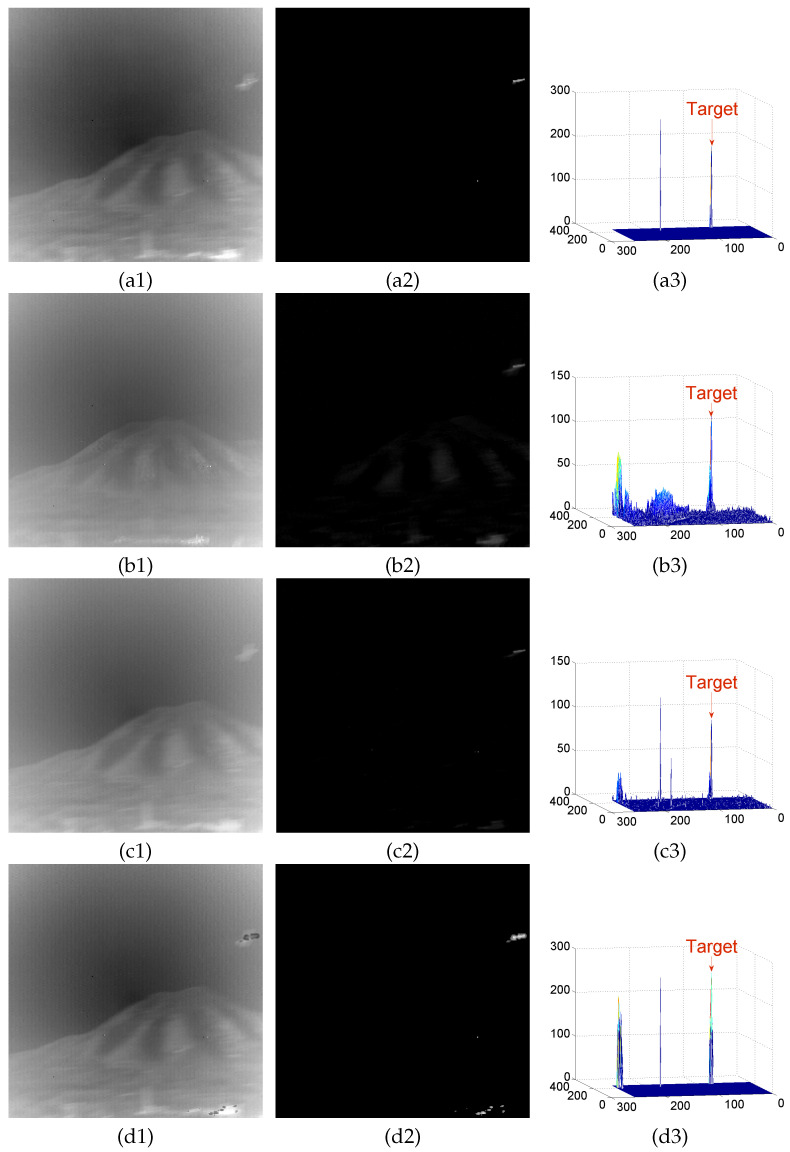

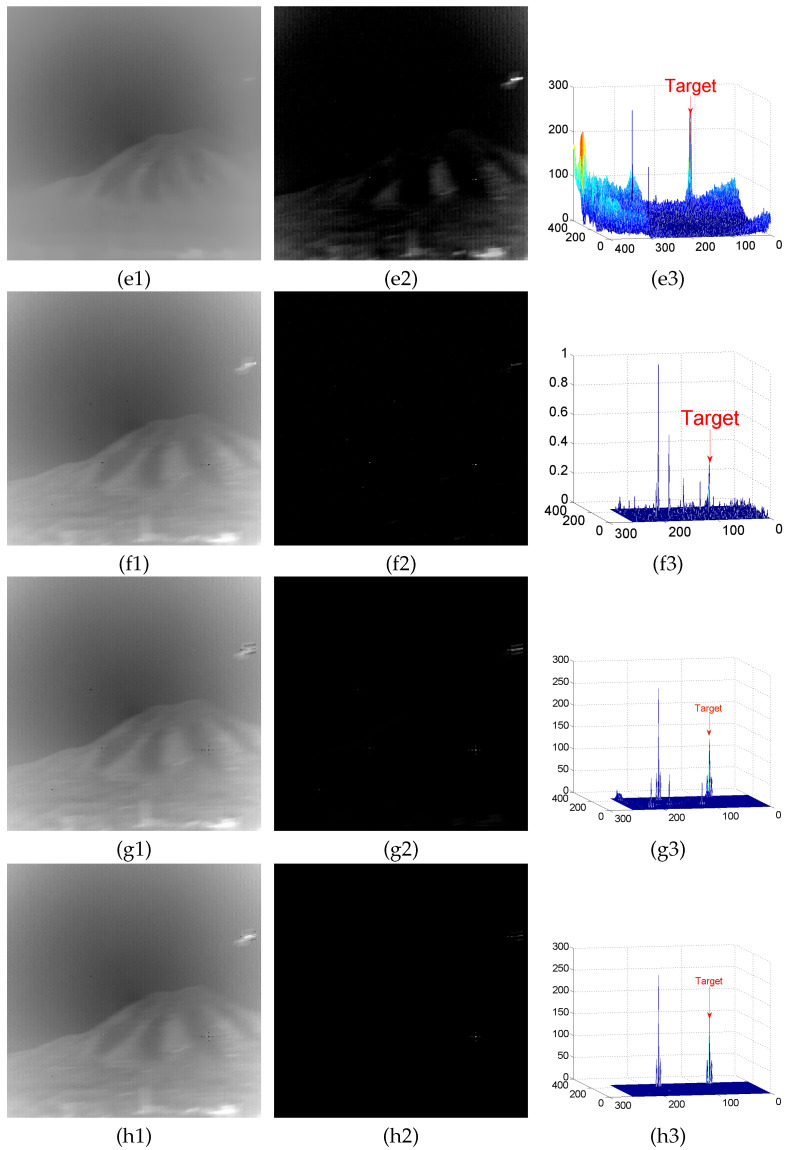

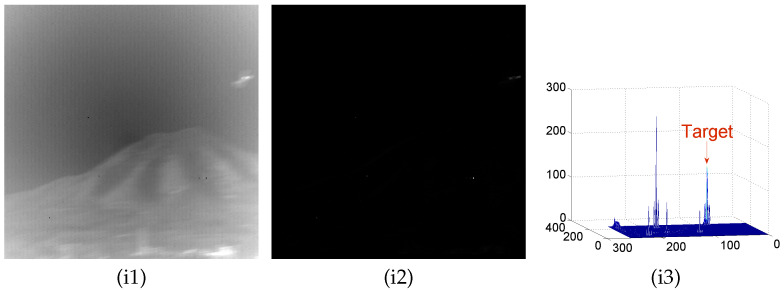
(**a**–**i**) denote the detection results of the PSTNN, RPCA, TV-PCP, VNTFR, ASTTV, SRWS, C2, C3, and proposed algorithm on sequence E, respectively, where (**a1**–**a3**) denote the background map, the differential map, and the 3D map of the obtained differential map, respectively.

**Figure A5:**
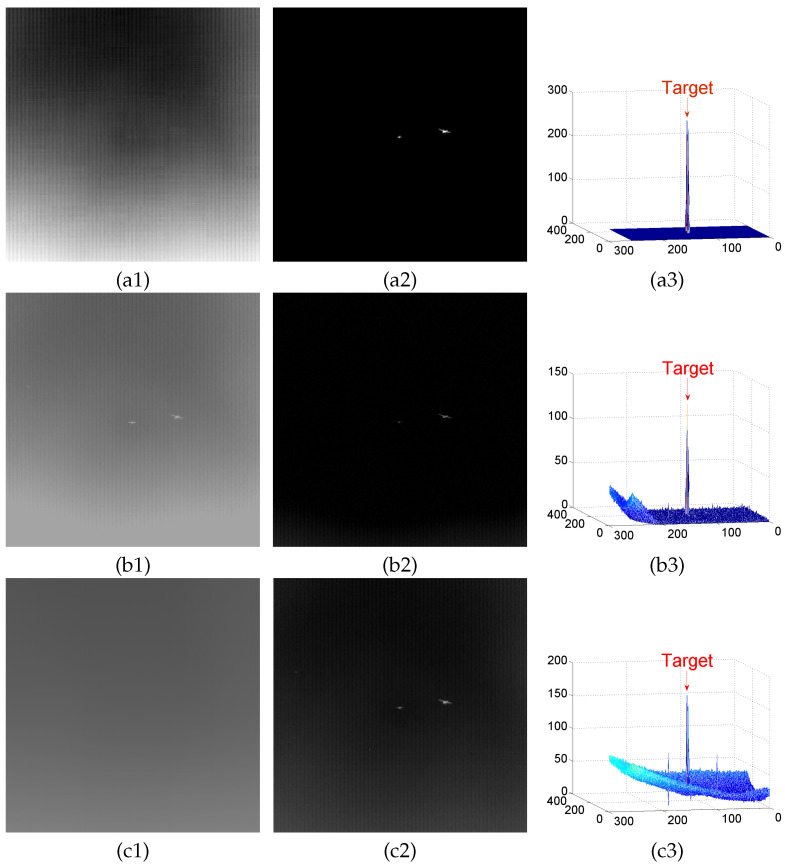

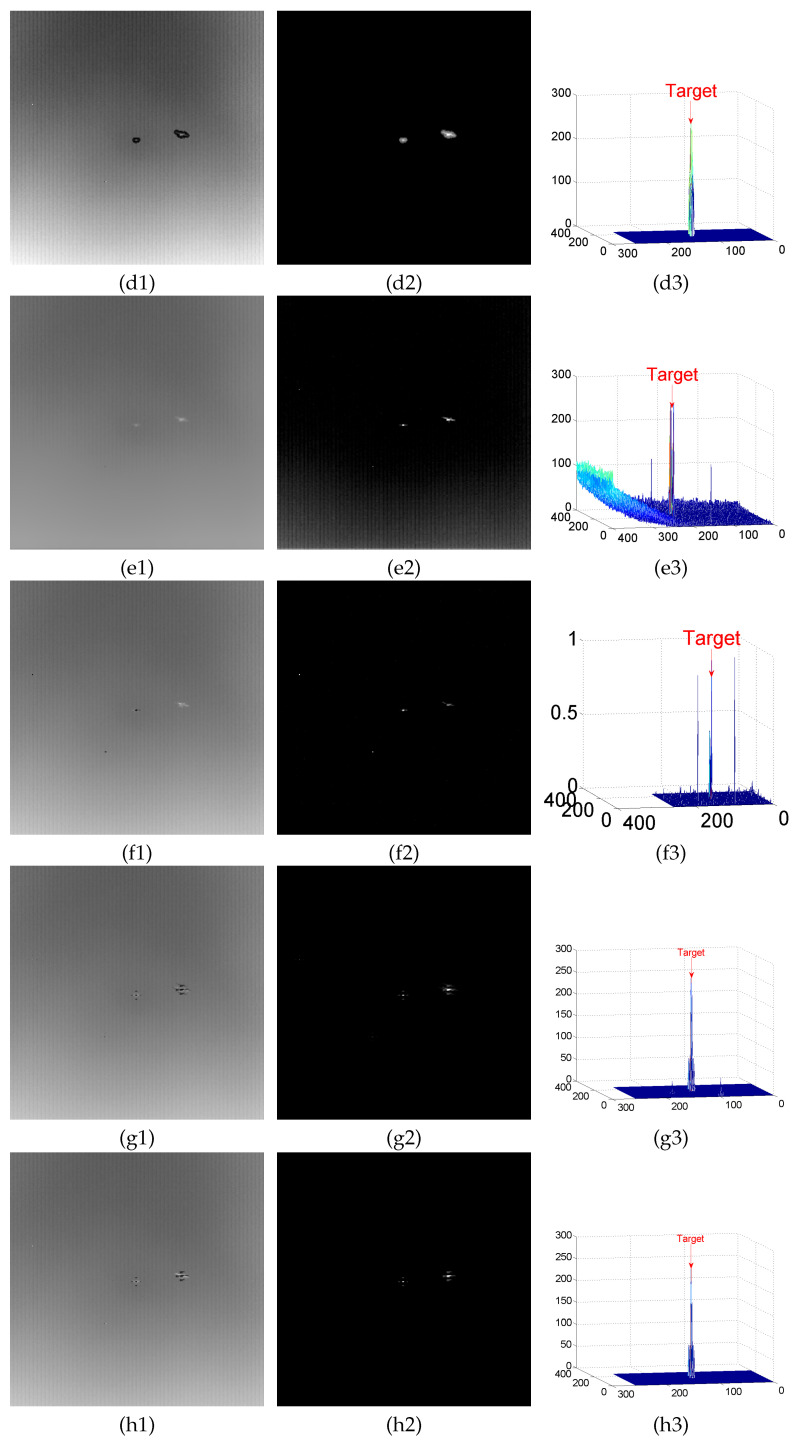

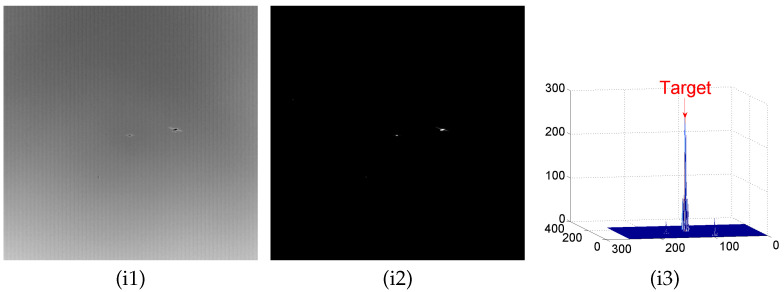
(**a**–**i**) denote the detection results of the PSTNN, RPCA, TV-PCP, VNTFR, ASTTV, SRWS, C2, C3, and proposed algorithm on sequence F, respectively, where (**a1**–**a3**) denote the background map, the differential map, and the 3D map of the obtained differential map, respectively.

**Figure A6:**
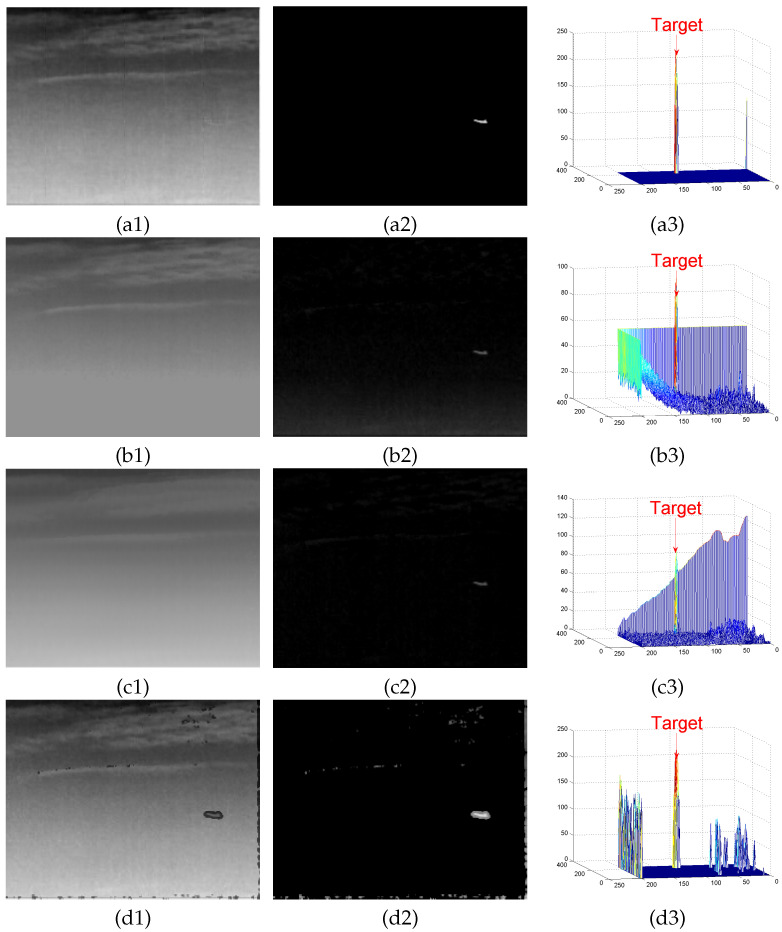

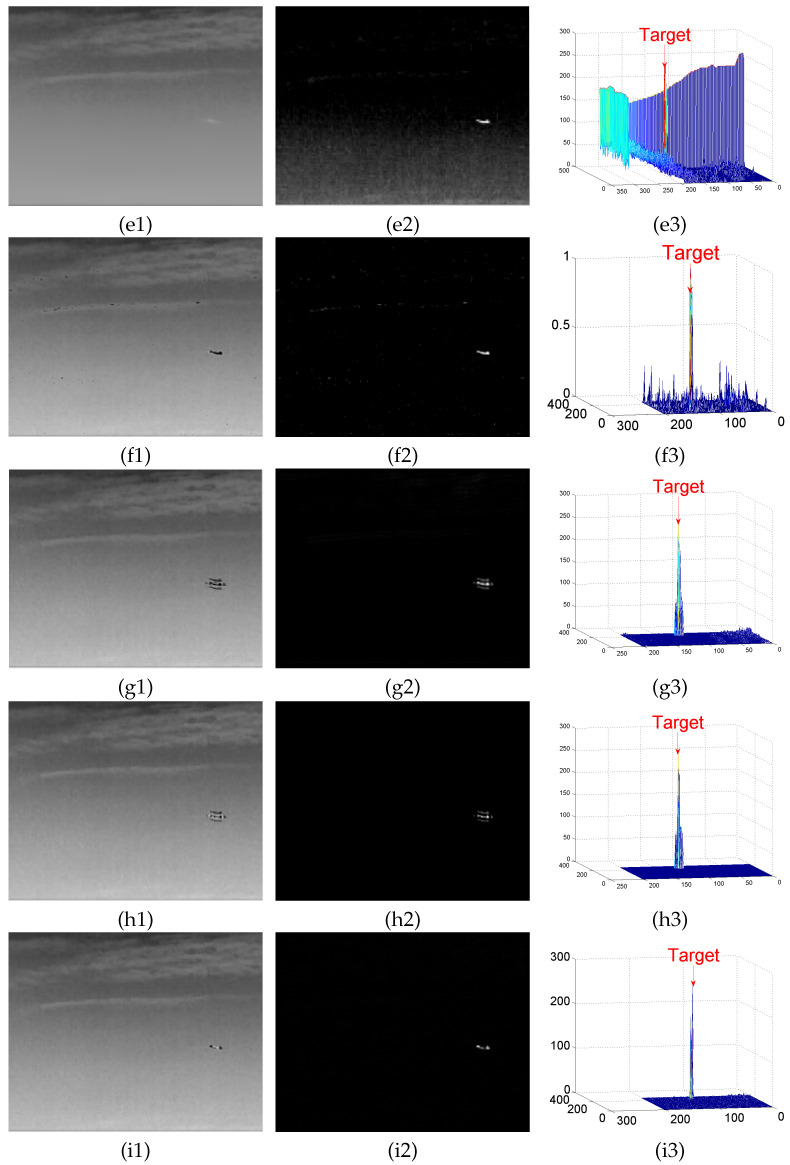
(**a**–**i**) denote the detection results of the PSTNN, RPCA, TV-PCP, VNTFR, ASTTV, SRWS, C2, C3, and proposed algorithm on sequence G, respectively, where (**a1**–**a3**) denote the background map, the differential map, and the 3D map of the obtained differential map, respectively.

**Figure A9:**
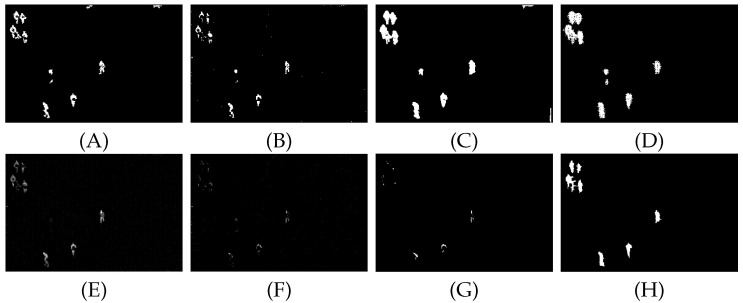
(**A**–**H**) shows the detection results of eight algorithms PSTNN, TV-PCP, VNTFR, anisotropy, ASTTV, SRWS, RPCA and propose method, respectively, under sequence C.

**Figure A11:**
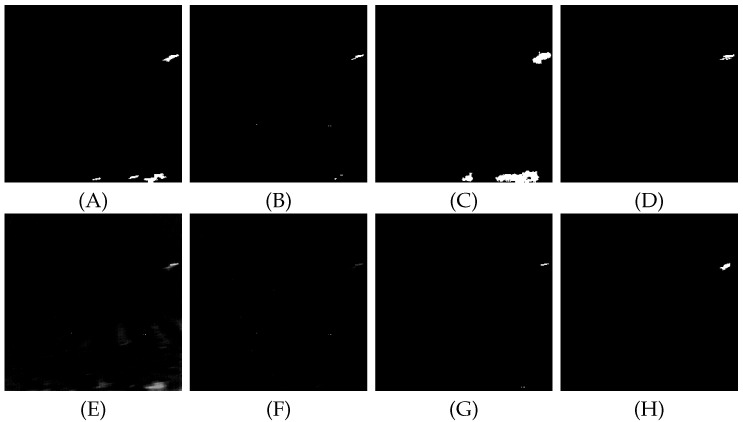
(**A**–**H**) shows the detection results of eight algorithms PSTNN, TV-PCP, VNTFR, anisotropy, ASTTV, SRWS, RPCA and propose method, respectively, under sequence E.

**Figure A13:**
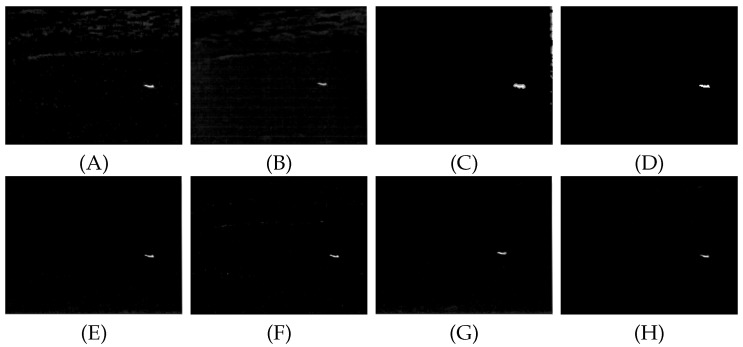
(**A**–**H**) shows the detection results of eight algorithms PSTNN, TV-PCP, VNTFR, anisotropy, ASTTV, SRWS, RPCA and propose method, respectively, under sequence G.

## Data Availability

The raw data support the findings of this study are openly available in OTCBVS thermal pedestrian database at https://download.csdn.net/download/bc727891259/9612819?locationNum=8&fps=1 (accessed on 25 August 2016). These raw images are public open source and can be used for free without copyright permission when not engaged in commercial use.

## Abbreviations

The following abbreviations are used in this manuscript:

VNTFR	Via Nonconvex Tensor Fibered Rank
PSTNN	Partial Sum of the Tensor Nuclear Norm
RPCA	Robust Principal Component Analysis
TV-PSMSV	Total Variation Partial Sum Minimization of Singular Values
STFM	Spatial-Temporal Features Measure
TV-PCP	Total Variation regulation and Principal Component Pursuit
SRWS	Self-Regularized Weighted Sparse
ASTTV	Asymmetric Spatial-Temporal Total Variation

## Appendix A. Experimental Results

### Appendix A.1. Results of Background Modeling Experiments

In this section, we show the background modeling results of the background modeling method proposed in this paper with the other seven algorithms for the remaining seven scenarios.

### Appendix A.2. Test Results

In this section, we show the target detection results of the algorithm in this paper with the other seven algorithms for the remaining seven scenarios.
